# KDIGO 2026 Clinical Practice Guideline for Anemia in Chronic Kidney Disease (CKD): a commentary from the European Renal Best Practice (ERBP)

**DOI:** 10.1093/ndt/gfag014

**Published:** 2026-01-28

**Authors:** Lucia Del Vecchio, Aleix Cases, Michele F Eisenga, Jolanta Małyszko, Jonathan Barratt, Davide Bolignano

**Affiliations:** Department of Nephrology and Dialysis, ASST Lariana, Como, Italy; Nephrology Unit, Hospital Clínic, Barcelona, Spain; Anaemia Working Group of the Spanish Society of Nephrology, Spain; Division of Nephrology, Department of Internal Medicine, University Medical Center Groningen, University of Groningen, Groningen, The Netherlands; Department of Nephrology, Dialysis and Internal Medicine, Medical University of Warsaw, Warsaw, Poland; Department for Cardiovascular Sciences, University of Leicester, Leicester, UK; Department of Medical and Surgical Sciences, University Magna Graecia of Catanzaro, Catanzaro, Italy

**Keywords:** anaemia, chronic kidney disease, erythropoiesis-stimulating agents, ESA hyporesponsiveness, HIF-PHI, iron

## Abstract

The 2026 Kidney Disease: Improving Global Outcomes (KDIGO) Clinical Practice Guideline for Anemia in Chronic Kidney Disease represents a substantial update, coming more than a decade since the previous KDIGO guideline on the topic. This time lapse was necessary in order to accumulate new scientific evidence that was meaningful for an updated guideline. The new guideline now includes updated recommendations and practice points for anaemia management, incorporating recent evidence on intravenous iron therapies and hypoxia-inducible factor prolyl hydroxylase inhibitors (HIF-PHIs). This European Renal Best Practice commentary critically evaluates and comments on relevant and newer recommendations and practice points, focusing on key aspects from a European perspective. The new guideline emphasizes the need for a comprehensive evaluation of the patient at the time of diagnosis, to identify additional causes of anaemia other than erythropoietin insufficiency. Coherently, the timing and the type of treatment should be individualized. Moreover, it introduces more proactive thresholds for intravenous iron supplementation, especially for haemodialysis patients. Erythropoiesis-stimulating agents are recommended as the preferred first-line therapy due to persisting concerns regarding cardiovascular safety and methodological limitations of available HIF-PHI trials. The commentary includes considerations on special aspects not considered by the new KDIGO guideline, such as pregnancy, gender-specific considerations, and interactions with sodium-glucose cotransporter 2 inhibitors or inflammation. The commentary also highlights regional regulatory differences between European and US drug approvals for HIF-PHIs. While supporting most recommendations, European nephrologists should consider local contexts, regulatory approvals and emerging evidence when implementing these guidelines in clinical practice.

## INTRODUCTION

Anaemia is one of the most prevalent and clinically relevant complications of chronic kidney disease (CKD), with increasing frequency and severity as glomerular filtration rate (GFR) declines. It is associated with substantial morbidity, including diminished functional capacity, reduced quality of life, and increased risks of cardiovascular events, CKD progression and mortality. Given the global burden of CKD and the growing number of individuals affected, optimizing anaemia management is a priority in nephrology practice and policy [1].

The 2026 Kidney Disease: Improving Global Outcomes (KDIGO) Clinical Practice Guideline for the Management of Anaemia in CKD [1] offers a long-awaited update to the 2012 recommendations [2]. At least part of the reason for the long delay in updating the Anemia Guideline was the need to accumulate new scientific evidence to justify an update. This extensively revised guideline incorporates new evidence, including data on the safety and efficacy of intravenous (IV) iron regimens since the publication of the FIND-CKD (A Randomized Trial of Intravenous Ferric Carboxymaltose Versus Oral Iron in Patients with Chronic Kidney Disease and Iron Deficiency) [3], REVOKE (A Randomized Trial to Evaluate Intravenous and Oral Iron in Chronic Kidney Disease) [4] and PIVOTAL (Proactive IV irOn Therapy in hemodiALysis patients) [5] trials. It also contains recommendations for the use of hypoxia-inducible factor prolyl hydroxylase inhibitors (HIF-PHI) following the publication of the extensive phase 3 programme for three of the front-runner molecules. These studies formed the basis for two Controversies Conferences organized by KDIGO in 2019 [6] and 2021 [7]. The Guideline also introduces revised terminology to better describe iron-restricted erythropoiesis and systemic iron deficiency (ID), and places strong emphasis on individualized, patient-centred care and shared decision-making.

Rather than summarizing the full guideline, this commentary of the European Renal Best Practice (ERBP) aims to critically appraise its most relevant innovations and implications from a European perspective. In the commentary, areas of uncertainty and different interpretations of the available evidence are highlighted, as well as implementation challenges in the European context and priorities for future research.

## DIAGNOSIS AND EVALUATION OF ANAEMIA IN CKD

Anaemia is defined as a reduction in one or more major red blood cell (RBC) indices obtained from the complete blood count (CBC), including haemoglobin (Hb) concentration, haematocrit (Hct) or RBC count. Among these, low Hb concentration and/or low Hct are the most commonly used parameters for the diagnosis of anaemia.

The new KDIGO guideline [1] has maintained the World Health Organization (WHO) criteria for anaemia in adults published in 1968 [8], defining it as a Hb <13 g/dL in males and <12 g/dL in females. The same values were used by the former KDIGO anaemia guideline from 2012 [2] and the new KDIGO CKD guideline from 2024 [9]. These Hb cutoffs were derived from a few European/North American datasets. We endorse the same values for anaemia definition. However, we point out that in 2024, the WHO reassessed Hb cutoffs using systematic reviews and multinational datasets [10]. While the classic adult cutoffs were maintained, refining was made for young children, pregnant women and people living at high altitude [10].

While both Hb and Hct are affected by volume shifts, Hct is especially dependent on plasma volume due to its calculation method. For this reason, Hb is a more reliable indicator than Hct in patients with CKD, who frequently experience changes in volume status. Notably, changes in plasma volume also occur when moving from the standing to the recumbent position (nearly a 10% increase), accompanied by inverse changes in Hct [11].

The anaemia of CKD is typically normocytic and normochromic. It is primarily due to a relative reduction in erythropoietin (EPO) synthesis by the kidney (a presumed reflection of the reduction in functioning kidney mass and disrupted renal oxygen sensing mechanism), shortened erythrocyte survival [12, 13], iron and other nutritional deficiencies (folate and vitamin B12), blood loss during haemodialysis (HD), uraemic toxin-induced inhibition of bone marrow response to EPO or systemic inflammation.

Anaemia is a common feature in many CKD patients, with prevalence rising together with estimated GFR (eGFR) decline, in particular when it falls below 30 mL/min/1.73 m^2^ [14, 15]. The diagnosis of anaemia in CKD should prompt an evaluation for alternative or additional causes [12, 13].

The basic set of measurements according to the new KDIGO guideline [1] includes CBC, reticulocytes, ferritin and transferrin saturation (TSAT). In contrast to the previous KDIGO recommendations, the new KDIGO Anemia Guideline [1] does not deliberately include vitamin B12 and folate assessment as part of the initial workup for anaemia in CKD [1]. The prevalence of vitamin B12 and folate deficiencies varies according to CKD stage, dietary patterns and medications (e.g. metformin) [16, 17]. Unlike vitamin B12, folate stores can decline during ESA treatment and are efficiently cleared by dialysis. Vitamin B12 can also be removed, particularly with high-flux dialysis techniques [18]. Furthermore, many B12-rich foods contain electrolyte concentrations that are inappropriate for patients on dialysis. Unfortunately, data on the prevalence of vitamin B12 deficiency in CKD and HD are scanty [19]. Nevertheless, a large percentage of the dialysis population is represented by old and frail patients, who are at risk for vitamin B12 deficiency, especially if hospitalized or in nursing home residency [20]. Polypharmacy, which is constantly present in HD or, more generally, in CKD patients, was found to be a significant risk factor [20]. Not to be forgotten, folate and vitamin B12 deficiencies are modifiable risk factors for cognitive impairment, a complication that represents a substantial burden in the HD population [21].

Altogether, we endorse the KDIGO recommendation that folate and vitamin B12 assessments should not be done in all patients at the diagnosis of anaemia. However, we stress the importance of testing folate and vitamin B12 in the presence of macrocytic anaemia, cognitive impairment, malnutrition, diabetes, advanced age and frailty.

Reticulocyte counts may further assist in distinguishing hypoproliferative anaemia from anaemia due to haemolysis or ongoing blood loss.

The suggested iron parameters continue to rely on serum ferritin levels and TSAT, despite the limitations of these measures of ID in the CKD setting [22, 23]. This differs from other guidelines recommending additional parameters, such as the percentage of hypochromic RBC or the reticulocyte Hb content [24]. The definitions of ID are similar to the previous anaemia guideline but with changes in their nomenclature: (i) a low TSAT and a low ferritin level [TSAT <20% and ferritin <100 ng/mL in non-dialysis dependent (NDD-CKD) or ferritin <200 ng/mL in CKD G5HD], previously named ‘absolute iron deficiency’, has changed to ‘systemic iron deficiency’; (ii) low TSAT and high ferritin level (ferritin >100–200 ng/mL with TSAT <20%) was previously termed ‘functional iron deficiency’ and it is now renamed as ‘iron-restricted erythropoiesis’ [1].

The new KDIGO guideline suggests that if the initial tests do not identify the underlying cause, further evaluation should be considered. This may include a peripheral blood smear, vitamin B12 and folate levels, liver enzymes, haptoglobin, faecal occult blood test, thyroid-stimulating hormone (TSH), lactate dehydrogenase, C-reactive protein (CRP), as well as serum protein electrophoresis with immunofixation, serum free light chains and urinary Bence-Jones protein.

We endorse the new KDIGO guideline recommending that CKD patients with anaemia and severe ID should be evaluated for blood loss with consideration of urology/gastroenterology/gynaecology referral. Malignancy should also be considered in the differential diagnosis of unexplained anaemia in patients with CKD.

## USE OF IRON TO TREAT IRON DEFICIENCY AND ANAEMIA IN CKD

The new KDIGO guideline on anaemia management in CKD [1] offers refined guidance on ID evaluation and iron therapy in the CKD setting. Table 1 compares the new KDIGO recommendations [1] with those given in 2012 [2] and with the ERBP position statement of 2013 [25].

**Table 1:** Comparison of KDIGO 2012, ERBP 2013 and KDIGO 2026 recommendations on iron therapy in CKD.

	KDIGO 2012 [2]	ERBP 2013 [25]	KDIGO 2026 [1]
Initiation criteria (NDD-CKD patients)	Trial of IV iron (or oral for 1–3 months) if:• TSAT ≤30% and ferritin ≤500 ng/mL when an increase in Hb without ESA is desired (2C)• OR an increase in Hb concentration or a decrease in ESA dose is desired in ESA treated patients (2C)	Trial of iron (oral first, if tolerated) if: • Absolute deficiency (TSAT <20% and ferritin <100 ng/mL) • OR increase in Hb desired with TSAT <25% and ferritin <200 ng/mL if an increase in Hb concentration without starting ESA treatment is desired • OR an increase in Hb concentration or a decrease in ESA dose is desired and TSAT is ≤30% and ferritin is ≤500 ng/mL	Suggest initiation of iron if: • Ferritin <100 ng/mL and TSAT <40% • OR ferritin 100–299 ng/mL and TSAT <25% (2D)
Initiation criteria (HD patients)	Trial of IV iron for 1–3 months if: • TSAT ≤30% and ferritin ≤500 ng/mL when an increase in Hb without ESA is desired (2C) • OR an increase in Hb concentration or a decrease in ESA dose is desired in ESA treated patients (2C)	Trial of IV iron if: • Absolute deficiency (TSAT <20% and ferritin <100 ng/mL) • OR increase in Hb desired with TSAT <25% and ferritin < 300 ng/mL if an increase in Hb concentration without starting ESA treatment is desired • OR an increase in Hb concentration or a decrease in ESA dose is desired and TSAT is ≤30% and ferritin is ≤500 ng/mL • In HD, IV iron therapy can be considered in those having higher serum ferritin levels and hyporesponsiveness to ESA or a risk/benefit ratio going against ESA use	IV iron if TSAT ≤30% and ferritin ≤500 ng/mL (2D) A proactive IV iron strategy suggested in HD (Practice Point 2.1)
Upper safety limits	Avoid iron administration if ferritin consistently >500 ng/mL (Not graded)	Do not intentionally exceed TSAT >30% or ferritin ≥500 ng/mL	Suggest withholding routine iron if ferritin ≥700 ng/mL or TSAT ≥40% (Practice Point 2.2) (Not graded)
Route of administration	NDD-CKD: route based on ID severity, venous access, prior response to oral iron, tolerance, patients’ compliance and cost (Not graded) IV iron in HD patients	Oral preferred in NDD-CKD (if tolerated); IV recommended in HD patients	In CKD not on HD: oral or IV based on patient factors (degree of anaemia and ID, CKD stage, patient preference, prior response, side effects, venous access, costs, availability of preparations (Not graded)
Monitoring	TSAT and ferritin every 3 months during ESA therapy (Not graded)	Similar approach, with caution at high ferritin levels	Reassessment of iron status emphasized: in NDD-CKD patients or CKD-5PD treated with iron monitor every 3 months; in HD patients every 1–3 months

PD, peritoneal dialysis; 2C/2D, grading of recommendations (2 = weak; C = low quality, D = very low quality).

In patients on HD, we endorse the initiation thresholds for iron therapy in patients on HD when ferritin is ≤500 ng/mL and TSAT is ≤30% (Recommendation 2.1), and we support the recommendation to prefer IV iron over oral iron (Recommendation 2.2). Additionally, based on the results of the PIVOTAL trial [5], we support the proactive administration of IV iron rather than a reactive approach (Practice Point 2.1). The PIVOTAL study demonstrated that proactive administration of high-dose iron sucrose (400 mg monthly, unless the ferritin concentration exceeded 700 µg/L or TSAT was ≥40%) was superior to reactive administration (0–400 mg monthly, triggered by ferritin <200 ng/mL or TSAT <20%) in terms of hard clinical endpoints and reduction of ESA doses or RBC transfusions, and was safe [5]. However, the PIVOTAL trial included incident HD patients (<1 year), therefore these results cannot be extrapolated to patients on HD for >1 year, or those with functional ID and inflammation, limiting its generalization to all HD patients. In addition, the findings do not apply to ESA-naïve patients, as ESA use was required for inclusion. Furthermore, patients with a short life expectancy, active malignancy, chronic liver disease or advanced heart failure (HF) (New York Hearth Association class IV), or those who were pregnant were excluded from the study. Finally, these results demonstrate the superiority of a proactive vs a reactive strategy at iron parameters indicating ID, but does not answer which is the optimal iron repletion strategy in this population and should be extrapolated with caution to other CKD populations, as discussed later.

Recommendation 2.3 proposes initiating iron therapy in NDD-CKD or peritoneal dialysis patients when ferritin is <100 ng/mL and TSAT <40%, or when ferritin is between 100–299 ng/mL with TSAT <25%. We support these thresholds, as they align with a more proactive iron strategy and represent a clear departure from the more conservative suggestions of the position paper of the ERBP of 2013 for the European population [25]. In fact, the FIND-CKD trial showed the superiority of IV iron targeting serum ferritin levels of 400–600 ng/mL unless TSAT was ≥40% in increasing Hb levels and serum ferritin vs oral iron, and reducing the need for other anaemia therapies, including ESAs [3]. Nevertheless, we would like to raise some concerns regarding the upper TSAT cut-off of 40%, which has been selected in alignment with ferritin <100 ng/mL. The optimal TSAT level for maximizing the Hb response to ESAs appears to be around 30% [26], casting doubt on the necessity of starting iron therapy in patients with ferritin just under 100 ng/mL and a TSAT, for example, of 35%. On the other hand, recent observational data suggest that TSAT levels between 30% and 40% may be associated with better clinical outcomes than levels around 30%. For instance, Nakai *et al*. [27] found that Japanese HD patients with TSAT between 30% and 40% experienced significantly fewer cardiovascular events than those with TSAT between 20% and 30%, particularly among those receiving long-acting ESAs. Similarly, Guedes *et al*. [28] demonstrated that ID, as captured by TSAT, is associated with higher risks of all-cause mortality and major adverse cardiovascular events (MACE) in NDD-CKD, regardless of the anaemic status [28]. A spline analysis indicated that the lowest risk occurred at a TSAT of 40%. These findings support initiating iron therapy in patients with TSAT <40%, particularly when serum ferritin is low. A small, short-term randomized controlled trial (RCT) of 46 HD patients, who were randomized to receive IV iron to a TSAT target of 20%–30% or 30%–50%, demonstrated a 30% decrease in ESA dose in the higher TSAT target group [29]. However, differences of mean TSAT across groups were low (27.2%–24.5%) in the control group vs (31.1%–34.9%) in the active group. Given that TSAT reflects iron availability, it is more acutely affected by recent iron administration than ferritin. In this respect, most observational studies do not report the timing of TSAT measurement relative to iron supplementation. For example, the FIND-CKD [3] trial discontinued oral iron one week before inclusion, but did not provide details on prior iron use in the enrolled population. In the opinion of the ERBP working group, these results are insufficient to support a target TSAT >40% which may perhaps expose patients to the risk of iron accumulation with time [29].

Despite the associations identified in observational studies being insufficient to infer clinical benefit or establish treatment thresholds, these findings are hypothesis-generating and require RCTs to define optimal TSAT values, both for increases in Hb and for hard clinical outcomes. Nevertheless, they are supported by a scientific rationale. Indeed, ID has been associated with an increase in platelet count and activity [30, 31], as well as with an increase in serum transferrin levels [32]. Recent studies have shown that increases in serum transferrin are associated with a prothrombotic phenotype by potentiating the FXIIa/thrombin axis and inhibiting antithrombin 3 [32, 33]. Hypoxia is also associated with increases in serum transferrin and a prothrombotic state [34]. However, the clinical relevance of these findings has been recently questioned in a non-CKD population [35]. Finally, ID increases fibroblast growth factor 23 (FGF-23) levels, which have been associated with worse outcomes in NDD-CKD patients and kidney transplant recipients (KTR) [36, 37].

With respect to the recommendation of prescribing iron therapy when serum ferritin is between 100 and 299 ng/mL with TSAT <25%, the authors acknowledge that this recommendation is based on the inclusion criteria in RCTs in ESA- or HIF-PHI-treated patients, in which the TSAT thresholds were higher than in ESA naïve patients (>20%), but adopted it to simplify the recommendations, acknowledging the lack of evidence to support it.

Another remark that we would like to make is that the systematic review underlying these recommendations included both dialysis and NDD-CKD patients, with the latter subgroup comprising a significant proportion of RCTs conducted in HF populations. These studies often evaluated cardiovascular outcomes rather than Hb response or other CKD-relevant endpoints (e.g. eGFR decline, reaching end-stage kidney disease), as shown in a recent metaanalysis not included in the guideline [38]. Most of the favourable outcomes from IV iron were seen in HF patients with reduced ejection fraction (HFrEF). In contrast, HF with preserved ejection fraction (HFpEF), which is more typical in CKD, is strongly underrepresented in the evidence base. The FAIR-HFpEF trial, designed to evaluate the effect of IV iron in this population with ID, was terminated early due to slow recruitment [39].

Overall, the thresholds proposed in Recommendation 2.3 for initiating iron therapy differ significantly from the more conservative ones in the ERBP position paper of 2013 for European populations [25]. In light of the current evidence, we endorse the updated KDIGO thresholds, while acknowledging the limitations discussed above and recognizing that more evidence is needed in the CKD population in this respect.

Recommendation 2.4, which leaves the choice between oral and IV iron to patient preference in NDD-CKD, is appropriate. But in case that a trial with oral iron is ineffective in increasing Hb levels after 1–3 months or in cases of intolerance, patients should be shifted to IV iron, as recommended in practice point 2.7.

Practice Point 2.2 suggests withholding iron in CKD patients when ferritin is ≥700 ng/mL or TSAT is ≥40% without differentiating CKD populations. These thresholds are primarily based on the PIVOTAL study protocol [5]. Given the outcomes of PIVOTAL and other trials in HD patients, these thresholds appear reasonable for that population. However, it is questionable whether this approach should be generalized to NDD-CKD patients without further supporting evidence. In addition, the PIVOTAL trial compared proactive and reactive approaches rather than specific ferritin or TSAT targets. Therefore, these results do not imply that maintaining ferritin levels between 200 and 700 ng/mL (e.g. 400 ng/mL) is inferior to the proactive strategy. In NDD-CKD, the largest relevant study is the FIND-CKD trial [3], which allowed a maximum ferritin level of 600 ng/mL and recommended withholding iron when TSAT was ≥40%. This RCT focused on intermediate markers with a relatively short follow-up to demonstrate long-term benefits. Hence, future RCTs should prioritize outcomes like fatigue, transfusion avoidance, hospitalizations and cardiovascular events. From a methodological point of view, targeting serum ferritin levels of 400–600 ng/mL seems reasonable in the NDD-CKD population, despite the above limitations.

We support Practice Points 2.3, which states that in people with CKD treated with oral iron, the choice between different formulations and dosing schedules is guided by cost, individual patient preference, tolerability and efficacy.

Newer oral iron formulations may offer improved efficacy and/or tolerability, but head-to-head RCT comparisons are minimal and the sample sizes and time of follow-up are limited to draw firm conclusions. Ferric citrate coordination complex is indicated for the treatment of concomitant elevated serum phosphorus and ID in adult CKD patients in the European Union. In a recent meta-analysis of six RCTs, ferric citrate reduced serum phosphorus levels and increased Hb, serum ferritin and TSAT, although it had a higher prevalence of gastrointestinal adverse events (AE) vs placebo [40].

Ferric maltol is an oral iron formulation also approved by the European Medicines Agency (EMA), characterized by high bioavailability, which may allow for effective iron repletion using lower daily doses. In a phase 3 trial involving NDD-CKD patients and ID anaemia, ferric maltol significantly improved Hb levels compared with placebo and was associated with increases in serum ferritin and TSAT, while maintaining a favourable tolerability profile. AE were mostly gastrointestinal in both groups but lead to treatment discontinuation in few patients [41].

Sucrosomial iron (not approved by the EMA) has been evaluated in NDD-CKD patients and IDA as compared with IV ferric gluconate. The proportion of patients who achieved ≥0.6 g/dL Hb increase at any time point between baseline and end of treatment (3 months) was significantly greater with IV ferric gluconate vs oral sucrosomial iron. Mean increases in serum ferritin were also significantly greater in the IV ferric gluconate group. Furthermore, after treatment discontinuation, Hb concentrations in the oral sucrosomial iron group returned to pretreatment values within 1 month after treatment discontinuation, but remained stable with IV ferric gluconate. Despite the incidence of AE was lower with sucrosomial iron, its effects on repletion of iron stores and on stability of Hb after drug discontinuation were lower [42]. Overall, the superiority in terms of efficacy and safety of the new oral formulations with respect to classical iron formulations requires further studies.

We also support practice point 2.4, which states that in people with CKD treated with IV iron, the choice between different formulations is guided by cost, individual preference and recommended dosing schedules. However, it is possibly too generic. Modern IV iron formulations [such as ferric carboxymaltose (FCM), ferric derisomaltose and ferumoxytol] offer the advantage of delivering high doses of iron over a short period of time, with an excellent safety profile and minimal release of labile iron. Ferumoxytol is not commercialized in the European Union. For Practice Point 2.4, we wish to emphasize that the choice of IV iron should not be based solely on cost, patient preference or dosing schedules, but also on specific clinical circumstances [6]. For example, the use of FCM in recently transplanted patients is not recommended, as these patients often have persistent hyperparathyroidism and are at high risk of developing severe hypophosphatemia due to FGF-23 excess following FCM administration [43]. In these instances, another IV iron compound known to cause less hypophosphataemia may be a more suitable option [44], or if FCM is administered, phosphate levels should be closely monitored.

Additionally, in European NDD-CKD, PD or KTR patients with anaemia and ID who are candidates for IV iron, a high-dose, low-frequency approach should be encouraged. This strategy takes into account not only the direct cost of IV iron formulations but also indirect costs (e.g. nursing and administrative expenses), the number of hospital visits and transportation needs, the importance of preserving vascular access sites, and the overall quality of life of patients [24].

Iron is essential for the proliferation of many pathogens—including bacteria and viruses—and may also affect immune function and host responses in ways that could enhance infection virulence. We fully endorse practice point 2.8 that recommends temporarily suspending iron therapy during systemic infection. Indeed, despite the fact that trials with IV iron (FIND-CKD [3], PIVOTAL [5]) and a systematic review did not demonstrate an increased risk of infections [45], the study protocols foresaw halting IV iron therapy during infection. Therefore, we support this practice point as a sign of prudence.

We also endorse Practice Point 2.11, which recommends considering iron therapy in patients with profound ID (ferritin <30 µg/mL and TSAT <20%) even in the absence of anaemia. While two small RCTs showed no benefit of IV iron in improving exercise capacity in non-anaemic NDD-CKD patients with ID [46, 47], a recent systematic review suggests that iron therapy may reduce the risk of hospitalization for HF and cardiovascular death, with consistent effects in both dialysis dependent (DD-CKD) and NDD-CKD populations [38]. However, these findings should be interpreted with caution, as much of the benefit was derived from NDD-CKD patients in HF trials rather than CKD-specific cohorts. Although some studies showed reductions in myocardial infarction, kidney failure and all-cause mortality, these effects were often imprecise, underpowered or driven by single studies. Furthermore, no consistent effects on eGFR decline or proteinuria have been demonstrated [3, 4]. Further studies are needed to show that the correction of ID with the cut-offs proposed in recommendation 2.3 are also beneficial, independent of anaemia. Until then, we agree with practice point 2.11 that profound systemic ID should be treated.

Restless legs syndrome (RLS) is more common in CKD, and especially in dialysis patients, than in the general population. ID has been suggested to play a role in this complication [48]. Three RCT with small sample sizes and a short follow-up reported improvements in clinical symptoms in HD and ID patients treated with IV iron vs placebo [49–51]. More recently, a metanalysis showed that IV FCM significantly improves RLS symptoms, especially in patients with ID [52]. Therefore, we suggest that in CKD patients with RLS, iron parameters should be measured and ID corrected with IV iron also in the absence of anaemia in selected cases, despite the limited evidence on the long-term results.

In patients with HFrEF and CKD, ID should also be corrected with IV iron according to the cardiology criteria of ID, due to the beneficial effects on clinical outcomes in these patients, independent of the presence of anaemia [53].

## USE OF ESAs, HIF-PHIs AND OTHER AGENTS TO TREAT ANAEMIA IN CKD

### Treatment initiation and ESA maintenance therapy

We fully endorse Practice Point 3.1.2 of the new KDIGO guideline, which suggests addressing all correctable causes of anaemia prior to initiating treatment with ESAs or HIF-PHIs [1]. Although this recommendation may seem self-evident, clinical practice too often reveals an automatic approach to anaemia treatment whereby physicians initiate ESA or HIF-PHI therapy without taking a holistic view of the patient. Treating or correcting other causes of anaemia could obviate the need for expensive treatments, such as ESAs and HIF-PHIs, along with their associated risks. In this regard, correcting ID and addressing causes of inflammation are particularly relevant, as these patients are more likely to be hyporesponsive to ESA or HIF-PHI therapy. Furthermore, ID during ESA or HIF-PHI therapy may increase the cardiovascular risk in CKD patients (see above).

Both ESA and HIF-PHI therapy increase iron demands through erythropoiesis stimulation, yet clinical trials typically provided similar or even lower iron supplementation to treatment groups compared with controls. This creates a potential scenario where ID—either present at treatment initiation or developing during therapy—could contribute to the elevated cardiovascular risk in vulnerable patients with these therapies [54].

We also fully endorse Practice Point 3.1.1, which suggests that the decision to use ESAs or HIF-PHIs to raise Hb should consider each individual’s symptoms, potential for harm from RBC transfusions and potential risk of AE (e.g. stroke, cardiovascular events, cancer) [1]. The decision should be shared with patients. This practice point emphasizes that ESAs and HIF-PHIs should not be automatically used to treat anaemia.

Practice Point 3.4.3.2 suggest monitoring Hb every 2–4 weeks during treatment with ESA or HIF-PHI and adjust the dose accordingly to avoid a rapid rise of >1.0 g/dL during that interval. This was meant for detecting rapid rises in Hb and preventing overshooting Hb targets. In our opinion, monitoring Hb every 2 weeks is unnecessary in most cases in everyday clinical practice, especially for NDD-CKD patients, also considering that early dose changes should be avoided as the practice point recommends. Furthermore, a rise in Hb >1 g/dL but lower than 2 g/dL at 4 weeks is considered adequate. Therefore, we consider more appropriate avoiding Hb increases >2 g/dL in 4 weeks as a more precise suggestion.

ESAs and HIF-PHIs are both effective agents for increasing Hb levels, as demonstrated by phase 3 RCT showing non-inferiority of HIF-PHIs versus ESAs [55–61] and superiority versus placebo [62–64]. Particularly for roxadustat, subjects experienced an initial sharper rise in Hb levels, possibly due to the protocol-chosen doses. This could be falsely interpreted as an advantage whereby patients reach Hb targets faster than those treated with ESAs. However, as previously mentioned, a sharp rise in Hb levels (>2 g/dL/month) either with ESAs or HIF-PHIs should generally be avoided, since it could be a possible reason for higher thrombotic risk or seizures in some patient subgroups (according to the SmPC of these drugs).

According to regulatory requirements of several health authorities, including the Food and Drug Administration (FDA) and the EMA, three HIF-PHIs underwent extensive, global, phase 3 clinical development. The majority of trials met the non-inferiority criteria for hard cardiovascular endpoints and safety parameters set by the FDA and EMA, with the exception of vadadustat in NDD-CKD patients [60]. Table 2 summarises the approval status and commercial a vaialbility of HIF-PHIs from FDA and EMA. Notably, regional differences were reported in this trial, with an excess number of MACE and non-cardiovascular deaths, largely from kidney failure, in the vadadustat group versus the darbepoetin alfa group in non-US/non-Europe regions [65]. Vadadustat was initially rejected by the FDA due to safety concerns but was subsequently approved for dialysis patients in 2024. Vadadustat is also approved by the EMA for dialysis patients, and it is commercially available in several European countries.

**Table 2:** Summary of the approval status and commercial availability of HIF-PHIs in Europe and the USA (updated to August 2025).

HIF-PHI agent	FDA	EMA	Commercial availability	Key safety concerns
Daprodustat	APPROVED For DD-CKD patients only; boxed warning for thrombotic risk	Application WITHDRAWN EMA recommended approval for DD-CKD patients; manufacturer withdrew application for commercial reasons	Europe: not available (withdrawn by manufacturer) USA: available for DD-CKD patients	Possibly higher cardiovascular and cancer risk in NDD-CKD on on-treatment analysis (with different follow-up periods between the two treatment groups)
Roxadustat	REJECTED Increased risk of death, thrombosis, and sepsis; methodological issues identified by FDA independent analysis	APPROVED For both NDD-CKD and DD-CKD patients	Europe: available for both NDD-CKD and DD-CKD patients USA: not available	Death; thrombosis; vascular access thrombosis; sepsis; methodological concerns in trial data; seizures
Vadadustat	APPROVED For DD-CKD patients only	APPROVED For DD-CKD patients only	Europe: available in several European countries for dialysis patients USA: available for DD-CKD patients	Thromboembolic events, hepatic toxicity, worsening of hypertension, seizures (minimal risk)

Last data verification 31 August 2025.

A prespecified on-treatment analysis of daprodustat in NDD-CKD patients also raised concerns about a higher relative risk of cancer-related AEs with daprodustat versus darbepoetin [59]. These findings could have possibly been influenced by bias risk estimation due to undercounting in the darbepoetin alfa group [66]. Moreover, on-treatment MACE analysis showed higher MACE incidence for daprodustat than darbepoetin alfa, possibly influenced by different dosing intervals and a 20% drug discontinuation rate before completing 1 year of follow-up in both groups. When examining regional differences, in Western Europe, the relative risk of MACE was consistently <1.0 in both DD- and NDD-CKD trials [67]. A secondary analysis of the ASCEND (Anaemia Studies in CKD: Erythropoiesis via a Novel PHI Daprodustat) trials also showed a non-statistically significant higher risk of hospitalization for HF in patients randomized to daprodustat [68]. Differences compared with darbepoetin alfa were more evident in NDD-CKD patients and in those with a history of HF. Daprodustat had not been approved for clinical use in NDD-CKD patients by the FDA and EMA. It received the FDA approval in February 2023 for DD-CKD patients only, with a boxed warning for thrombotic risk. Despite the EMA’s recommendation for approval in DD-CKD, the manufacturer withdrew its European application in July 2023.

Concerns were also raised by the FDA regarding roxadustat due to increased risk of death, thrombosis and sepsis, leading to denial of its approval. Moreover, the FDA highlighted several methodological issues following an independent analysis of submitted roxadustat trial data [69]. Conversely, roxadustat is approved for clinical use by the EMA in both NDD-CKD and DD-CKD patients. The EMA concluded that cardiovascular and mortality risks appeared to be similar to ESA based on data from the ‘Hb correction studies’ in NDD-CKD and DD-CKD patients and considered that evaluation in other data pools (including comparison with placebo and in stable dialysis patients) are associated with methodological and study design issues that complicate interpretation.

These data indicate a complex situation regarding cardiovascular safety with HIF-PHIs, ranging from potential protection to neutral effects to potential harm, even for the same agent, without clear explanation. Moreover, in recent years, several meta-analyses have shown overall similar safety data for HIF-PHIs compared with ESAs [70–74], while others found opposing findings [75].

While we recognize the rationale behind KDIGO recommendation 3.1.1 favouring ESAs as first-line therapy for anaemia treatment [1], we believe the European context warrants additional consideration. The KDIGO guideline assigned this recommendation a grade of 2D, reflecting acknowledged limitations including wide confidence intervals for MACE outcomes in meta-analyses, trial exclusions that reduced real-world applicability, and concerns regarding risk of bias, inconsistency and indirectness of the evidence. We also fully acknowledge that ESAs benefit from decades of clinical experience and more established safety profiles, advantages that HIF-PHIs do not yet possess. The concerns raised regarding some HIF-PHI trials, including safety signals and methodological limitations, are valid considerations. However, given that EMA has approved some HIF-PHIs drugs based on their regulatory assessment, and considering the non-inferiority demonstrated for Hb response and safety in the phase 3 RCTs of these molecules, we suggest that in the European context, both ESAs and EMA-approved HIF-PHIs could be considered as treatment options for anaemia in CKD patients, with ESAs maintaining their established role as the reference standard while HIF-PHIs represent an alternative when clinically appropriate.

Additionally, the potential for drug–drug interactions with HIF-PHIs should be carefully considered since CKD patients typically receive multiple medications, whereas ESAs as biological agents have a lower propensity for pharmacokinetic interactions. The consideration of HIF-PHIs as an alternative option does not apply to selected patient categories (see below for safety of HIF-PHI in special populations) in whom HIF-PHIs should be used with extreme caution or completely avoided [1, 76].

Table 3 summarizes different suggestions for HIF-PHIs use in CKD according to the 2026 guideline [1] and to the present commentary. Interestingly, most of these concerns are actually equally applicable to the use of ESAs [77]. For balance, a table including concerns on ESAs should have been included in the KDIGO guideline [1].

**Table 3:** KDIGO and ERBP suggestions for ESA and HIF-PHI use in CKD anaemia.

Category	KDIGO guideline	ERBP suggestions
ESAs		
CONTRAINDICATIONS/AVOID or	• Avoid or lower Hb target for high-risk patients (recent cardiovascular event/thromboembolism, active cancer with curative intention) • Suspend during hospitalization for acute stroke, vascular access thrombosis, or thromboembolic events; individualize re-initiation • PRCA after any ESA	• Stroke risk and malignancy are not absolute contraindications; weigh risk/benefit • Suspend during hospitalization for acute stroke, vascular access thrombosis, or thromboembolic events; individualize re-initiation • Avoid in active cancer with curative intention • Uncontrolled hypertension • PRCA after any ESA
USE WITH CAUTION	Shared decision-making in cancer/previous stroke	• Lower Hb target for high-risk patients • Vascular access with a high risk of thrombotic complication • Seizures • Pregnancy
CONSIDER USE	In CKD-G5D at Hb 9–10 g/dL; in NDD-CKD individualize if Hb <10 g/dL. Maintain <11.5 g/dL. Use lowest effective dose	•NDD-CKD: do not allow Hb <10 g/dL; consider earlier start (<11 g/dL) in symptomatic IHD/low risk. Maintain around 10–12 g/dL • CKD-G5D: do not allow Hb <10 g/dL. Maintain around 10–12 g/dL • Patient with low compliance for oral treatment or high pill burden
HIF-PHIs		
CONTRAINDICATIONS/AVOID	• Active cancer or with a history of cancer not in complete remission for at least 2–5 years (based on trial exclusion criteria) • Polycystic kidney disease • Proliferative retinal disease • Pulmonary arterial hypertension • Pregnancy	• Patient with a cardiovascular or thrombotic event in the previous 3 months • History of malignancy in the last 5 years • Polycystic kidney disease • Untreated proliferative diabetic retinopathy, macular degeneration and retinal vein occlusion • Idiopathic pulmonary arterial hypertension • Pregnancy
USE WITH CAUTION	• Prior cardiovascular events (i.e. stroke, myocardial infarction) • Prior thromboembolic events (i.e. deep vein thrombosis, pulmonary embolism) • Prior vascular access thrombosis • Hepatic impairment • Seizures, exfoliative dermatitis, hypothyroidism, bacterial infections/sepsis (roxadustat)	• Vascular access with a high risk of thrombotic complication • Retinal disorders • Autoimmune diseases • History of cured malignancy or without recurrence for at least 5 years • Kidney transplant recipients • Hypothyroidism (roxadustat) • Hepatic impairment • Seizures • Drug–drug interactions
INSUFFICIENT DATA	Assessment; dedicated studies needed: • Post-kidney transplant anaemia • Children	Assessment; dedicated studies needed: • Children
CONSIDER USE	Not specified	• Patient preference for oral treatment (accessibility, convenience, ease of administration, no storage requirements) • Challenges to starting or receiving ESAs (needle-phobia, unable to self-administer ESAs) • Challenges to administering iron therapy or when increased iron availability is desired • ESA hyporesponsiveness or intolerance • Chronic inflammatory states (CRP ≥3 mg/L) • Home HD • Peritoneal dialysis • Hypersensitivity or unavailability of IV iron • After recovery of ESA-related PRCA

The new KDIGO Anemia Guideline maintains the same Hb thresholds for initiating ESA therapy established in 2012 [2]. Recommendation 3.2.1 specifies initiating ESA when Hb falls to ≤9.0–10.0 g/dL (90–100 g/L) for patients on HD or PD (2D) [1]. Recommendation 3.2.2 emphasizes that for NDD-CKD patients, including KTR and children, the decision to start ESA therapy should be individualized based on anaemia-related symptoms, potential benefits of higher Hb levels, and risks associated with RBC transfusion or ESA treatment (2D) [1]. The guidelines also maintain the same Hb target during maintenance ESA therapy, recommending levels below 11.5 g/dL (1D) [1]. In this respect, we support the same observations and thus the same suggestions for ESA start and Hb targets for maintenance made by ERBP in 2013 [25]. As stated in the ERBP 2013 position statement [25], evidence from both TREAT [78] and other studies (including a meta-analysis showing higher left ventricular mass index in patients with Hb <10 g/dL and its regression while increasing Hb with ESA [79]), suggests that timely anaemia correction, particularly in more advanced CKD, may confer cardiovascular and renal benefits, and we therefore feel that Hb levels should not routinely fall below 10 g/dL in both NDD- and DD-CKD patients. For maintenance therapy, ERBP suggested using ESA to maintain Hb values between 10 and 12 g/dL, with individualization within this range based on patient comorbidities [25]. Notably, KTR may possibly benefit of higher Hb levels in the perspective of preserving graft function without safety concerns [80–84]. Nevertheless, we endorse the KDIGO statement that the decision and timing to start or not ESA therapy should be individualized according to individual patient characteristics and clinical preferences. Given that phase 3 clinical trials of HIF-PHIs were designed to target conventional Hb levels comparable to those used with ESAs, we support the recommendation that there is insufficient evidence to suggest different Hb thresholds and targets for HIF-PHI therapy [1].

Considering the thrombotic risks associated with both ESAs and HIF-PHIs, we also endorse the guideline’s suggestion to suspend ESA therapy during hospitalization for acute stroke, vascular access thrombosis, or thromboembolic events (Practice Point 3.4.3.4) [1]. In our view, this precautionary approach should similarly apply to HIF-PHI treatment given the comparable thrombotic risk profile of these agents.

In the last decade, no significant new evidence has emerged regarding the optimal administration frequency for different ESA molecules. The 2026 KDIGO guideline suggests to individualize the frequency of administration based on patient preferences, local practice and type of ESA [1]. However, administration frequency is left quite wide for short-acting ESAs compared with methyl polyethylene glycol-epoetin beta (the ESA molecule with the longest half-life). Indeed, the same frequency of administration is recommended for methoxy polyethylene glycol-epoetin beta (every 2 weeks for dialysis patients at initiation), as for epoetin alfa initial dose for NDD-CKD patients despite the markedly different half-lives of these two molecules. Moreover, no information is given on the frequency of administration during maintenance therapy. According to several studies, stable maintenance NDD-CKD patients could potentially receive ESAs subcutaneously (SC) up to every 4 weeks. However, specifically for epoetin alfa and darbepoetin alfa, this approach is particularly suitable for patients with lower dose requirements. For those requiring higher doses, unnecessarily prolonged administration intervals necessitate higher ESA doses (dose penalty) to achieve the same Hb increase and expose patients to elevated ESA dose peaks. While we acknowledge that administration frequency should consider patient convenience and CKD stage (dialysis versus non-dialysis), we emphasize that dosing decisions should also account for treatment phase (initiation versus maintenance), individual dose requirements (delayed administration frequency is not recommended for hyporesponsive patients), and the pharmacokinetics and pharmacodynamics of individual agents with their corresponding optimal administration schedules.

While the 2026 KDIGO guideline allows flexibility in ESA administration route, it does not take into account different dosing for SC versus IV ESAs—even at higher dose levels [1]. Unlike short-acting ESAs, long-acting agents (darbepoetin alfa or methoxy polyethylene glycol-epoetin beta) exhibit comparable efficacy at equivalent doses regardless of administration route [85]. In our opinion, this should be considered when high ESA doses are required, particularly in HD patients receiving IV therapy.

Table 4 summarizes indications on administration frequency of 2012 [2] and 2026 KDIGO guideline [1].

**Table 4:** Comparison of KDIGO 2012 and 2026 guidelines on ESA administration frequency.

	KDIGO 2012 guideline [2]	KDIGO 2026 guideline [1]
Initial ESA dosing frequency	Not specified in detail; administration route and frequency should be individualized (SC or IV) based on patient’s factors	Individualize the frequency of administration based on patient preferences and type of ESA (up to two weeks for epoetin alfa and up to once a month for darbepoetin alfa and methoxy polyethylene glycol-epoetin beta in NDD-CKD, up to two weeks for darbepoetin alfa and every two weeks for methoxy polyethylene glycol-epoetin beta in DD-CKD; Table 7)
Maintenance ESA dosing frequency	Tailor dose and frequency to achieve and maintain Hb within target range. Adjustment guided by clinical response and Hb trends	Not specified that the frequency could be delayed for every ESA molecule
Use of long-acting ESAs	Recognized but no specific frequency mentioned	Methoxy polyethylene glycol-epoetin beta possibly at reduced administration frequency compared with the other agents with a shorter half-life
Flexibility of administration	Emphasis on individualization, particularly for route (SC vs IV) and frequency	Mostly based on patient preferences and local practice. Higher doses of epoetin required when administered via IV as compared with SC for short-acting ESA, which in turn will increase costs; no differences in dose recommendations for IV versus SC darbepoetin alfa
Considerations in NDD- CKD vs DD-CKD	No clear differentiation in frequency recommendations between NDD-CKD and DD-CKD patients	Less frequent dosing possible particularly in NDD-CKD to facilitate outpatient management
Overall strategy	ESA dose and frequency should be titrated based on Hb response and clinical status	Patient preferences and local practice patterns often determine the choice of ESA and the frequency of administration

### HIF-PHI treatment initiation and maintenance

The initiation and maintenance dosing of HIF-PHIs follow exactly the same clinical paradigm as for ESAs and, in clinical practice, mean very little change in current clinical pathways for the management of anaemia of CKD. As with ESAs, the 2026 KDIGO guideline recommends that before initiating a HIF-PHI all correctable causes of anaemia are addressed (Practice Point 3.1.2). Target iron parameters prior to initiation are the same as those expected before initiation of ESA (Recommendation 2.1 and Recommendation 2.3).

Many HIF-PHI studies have documented decreased ferritin and hepcidin levels during treatment, indicating iron depletion [86]. Although from a theoretical standpoint, HIF-PHIs increase iron absorption and availability, data from clinical trials and meta-analyses indicate that compared with ESA, patients receiving HIF-PHI require less IV iron therapy [87]. However, this difference is not clinically meaningful, particularly considering that iron protocols were not standardized and equalized between control and active treatment groups. For this reason, we confirm the suggestion of the ERBP commentary on HIF-PHIs [76] that these drugs should be initiated only when iron stores have been replenished.

The KDIGO guideline suggests that the same Hb thresholds for the initiation and maintenance of HIF-PHIs are used as those recommended or suggested for ESA therapy (Practice Point 3.5.2) [1]. Not surprisingly, and again as with ESAs, HIF-PHIs should be initiated at the recommended starting doses (Practice Point 3.5.3) and, as with any drug used in clinical practice, including ESAs, the dose prescribed should be the lowest to achieve the desired clinical benefit and the final dose prescribed should not exceed the recommended maximum dose (Practice Points 3.5.4 and 3.5.5). Importantly, ESAs and HIF-PHIs should not be used in combination, including in the setting of ESA hyporesponsiveness, due to the lack of any data on safety and efficacy of this combination (Practice Point 3.5.1).

### HIF-PHI monitoring

Monitoring patients taking HIF-PHI is no different from the labelled recommendations for monitoring of patients on ESAs, again meaning that there is little change in clinical pathways when HIF-PHIs are used. Similarly, frequency of monitoring of iron parameters is no different from those treated with ESAs and iron (Practice Point 2.5). Just as with ESAs, treatment with HIF-PHIs should be suspended in those who experience cardiovascular events (e.g. stroke, myocardial infarction); thromboembolic events (e.g. deep vein thrombosis, pulmonary embolism); vascular access thrombosis; or newly diagnosed cancer (Practice Point 3.6.3). Again, entirely consistent with the advice for ESAs, HIF-PHIs should be discontinued after 3–4 months if a desired erythropoietic response has not been achieved (Practice Point 3.6.3).

While the monitoring of patients on HIF-PHIs is in practice no different from those on ESAs, there is one additional requirement for those patients treated with roxadustat. Cases of central hypothyroidism have been reported in cohorts from Japan and China, believed to be due to roxadustat binding to the thyroid hormone receptor β (THRβ) amplifying the negative feedback loop in the hypothalamic–pituitary–thyroid axis [88, 89]. As such, it is advised that thyroid stimulating hormone and free T3 and T4 are measured after 4 weeks of therapy initiation.

### ESA hyporesponsiveness

ESA hyporesponsiveness is defined as the inability to achieve the target Hb level using higher than usual doses of ESAs or increasingly higher requirements of ESAs doses to maintain a target Hb level [90, 91]. ESA hyporesponsiveness has been associated with an increased risk of MACE [92–94].

However, there is no consensus on the definition of ESA hyporesponsiveness across institutions, such as KDOQI (Kidney Disease Outcomes Quality Initiative), KDIGO and ERBP. The different recommendations are summarized in Table 5.

**Table 5:** Definitions of ESA hyporesponsiveness in CKD across different guidelines.

Source	Year	Definition	ESA dose thresholds	Notes
EBPG [97]	2004	Failure to attain or maintain target Hb with: • ≥300 IU/kg/week of epoetin, or • ≥1.5 µg/kg/week of darbepoetin alfa	∼20 000 IU/week epoetin or ∼100 µg/week darbepoetin	Based on fixed dosing thresholds
KDOQI [96]	2006	Failure to achieve or maintain target Hb despite: • ≥450 IU/kg/week of IV epoetin, or • ≥300 IU/kg/week of SC epoetin	≥450 IU/kg/week (IV) or ≥300 IU/kg/week (SC)	No threshold for darbepoetin; fixed high-dose approach
KDIGO [2]	2012	Initial hyporesponsiveness: no Hb increase after 1 month of appropriate ESA dosing Acquired hyporesponsiveness: ≥2 increases in ESA dose (up to 50% above stable dose) to maintain Hb	No fixed dose threshold	Focus on relative response; discouraged repeated dose escalation
NICE (UK) [95]	2015	ESA hyporesponsiveness suspected if: • Failure to reach Hb target despite optimal iron and ESA dosing • Use of high ESA doses without adequate Hb response	No specific thresholds; judgment-based	Used in practice and health technology assessments; conservative estimate of prevalence
UK Kidney Association [24]	2025	• Failure to reach the target Hb despite SC epoetin dose > 300 IU/kg/week (450 IU/kg/week IV epoetin), or darbepoetin dose > 1.5 µg/kg/week, or equivalent dose of methoxy ethylene glycol epoetin beta	See left column	Consider: • Accepting lower Hb target • Alternative trial with HIF-PHI
KDIGO [1]	2026	• No clear definition: not achieve target Hb levels despite a significant increase in ESA doses or continue to require high doses to maintain the target	No fixed dose threshold; focuses on relative increase and clinical reassessment	• Focus on relative response; • Avoid excessive ESA dosing and escalation • Investigate reversible causes • Consider HIF-PHIs in selected patients

According to KDOQI, ESA hyporesponsiveness is defined as failure to achieve or maintain a desired Hb concentration using a maximum dose of 450 units/kg per week of IV epoetin or 300 units/kg per week of subcutaneous epoetin [96]. No criteria were provided for darbepoetin alfa. According to package inserts, 45 000 units/week of epoetin corresponds roughly to 100 µg/week of darbepoetin alfa.

In 2012, KDIGO defined ESA hyporesponsiveness as having no increase in Hb concentration from baseline after the first month of ESA treatment on appropriate weight-based dosing [2]. It also classified patients as having acquired ESA hyporesponsiveness if, following treatment with stable ESA doses, they required two dose increases of up to 50% above the previously stable dose in order to maintain a stable Hb concentration [2]. ERBP defined ESA hyporesponsiveness as failure to attain or maintain desired Hb concentration with 300 units/kg per week of epoetin (approximately 20 000 units/week) and 1.5 µg/kg per week of darbepoetin alfa (approximately 100 µg/week) [97]. Use of ESAs depends upon the local policy, dialysis modality (PD vs HD) and reimbursement, therefore from the practical point of view, ESA resistance may be considered when patients require greater doses of ESA than 90% of patients in the dialysis unit. ESA hyporesponsiveness can be acute or chronic (>4 months) and its prevalence varies by geographical regions [6, 98].

Atzinger *et al*. [99] evaluated the prevalence of ESA hyporesponsiveness in a real-world setting using the Fresenius Medical Care (FME) EuCliD database across Europe and South Africa. A total of 85 259 out of 380 675 CKD patients receiving KRT had a weak response to ESA therapy. Depending on the criteria used [KDIGO, National Institute for Health and Care Excellence (NICE), erythropoiesis resistance index and clinical practicality algorithm (CPA)], the prevalence of ESA hyporesponsiveness varied greatly, from 5.2% (NICE) to 47.9% (CPA). PD patients appeared to have lower prevalence of ESA hyporesponsiveness; however, data are very limited [99].

As in 2012, the new KDIGO guideline confirms the definition of initial ESA hyporesponsiveness and acquired ESA hyporesponsiveness, without fixed absolute dose threshold for hyporesponsiveness but rather emphasizing relative increases from a previously effective dose. The new KDIGO guideline also confirms the importance of avoiding repeated escalation of ESA doses in non-responders. If no reversible cause is identified, clinicians are advised to reassess the indication for ESA therapy, avoid excessive ESA exposure and consider alternatives (e.g. iron optimization, HIF-PHI in selected cases).

In this respect, HIF-PHIs may have a theoretical advantage over ESAs as they promote hypoxia-induced expression of EPO while suppressing hepcidin and ferritin, and possibly other pro-inflammatory cytokines [85, 86, 100]. However, some data showed the opposite [101], underlying the complex interplay of the HIF system on inflammation. According to some secondary analyses of phase 3 RCTs, they could be effective in increasing Hb levels in patients with increased inflammatory markers while avoiding dose increases [102, 103]. However, the clinical effect looks mild. Moreover, true hyporesponsive patients or those with significant inflammation were excluded from these RCTs.

In Practice Points 3.7.2 to 3.7.4, the new KDIGO guideline recommends a 3- to 4-month trial of a HIF-PHI in ESA-hyporesponsive patients, if there is a clinical indication to increase Hb levels, avoid transfusion or improve anaemia-related symptoms. They also propose to use the lowest possible dose to alleviate anaemia-related symptoms and/or reduce the risk of RBC transfusions and discontinue the treatment if the desired erythropoietic response is not achieved; given the cardiovascular safety concerns raised in large global cardiovascular safety trials, HIF-PHI use in people with CKD and ESA hyporesponsiveness may further increase their pre-existing risk for serious cardiovascular events [1]. The limited evidence of these recommendations is acknowledged by several authors [104–107]. Nevertheless, data from a secondary analysis of the ASCEND-D trial did not show evidence for differential associations of baseline ESA resistance and risk of MACE by randomization to either daprodustat or conventional ESAs [92]. We endorse these recommendations but also back the recommendation to examine the effects of HIF-PHI in ESA hyporesponsive patients (DD- and NDD-CKD) on critical and important endpoints. Given the limited available evidence, in our view, the decision to continue or discontinue HIF-PHI therapy should be individualized based on single patient response. While the 2026 KDIGO guideline suggests discontinuing HIF-PHI after 3 months in non-responsive patients due to potential cardiovascular concerns [1], this timeframe may be considered relatively short and lacks robust supporting evidence for such a definitive approach.

Conversely, we endorse practice points 3.7.1 to identify and treat the underlying causes of ESA hyporesponsiveness if possible, acknowledging that in around 30% of cases no cause can be identified. The two most common causes of ESA hyporesponsiveness are ID and/or inflammation. At first, absolute and/or functional ID should be excluded. Persistent inflammation is common in CKD [108], which may stimulate hepcidin synthesis, which in turn downregulates the iron exporter, ferroportin, resulting in iron-restricted erythropoiesis [109]. Other common causes of chronic inflammation in DD-CKD patients such as catheters, skin/wound infections, failed graft, occult infection of an old, nonfunctioning arteriovenous graft should be evaluated. Inadequate dialysis adequacy may also contribute to the inappropriate response to ESA. Relatively uncommon causes associated with ESA hyporesponsiveness include severe hyperparathyroidism with osteitis fibrosis cystica, malignancies, haematologic bone marrow disorders, such as myelodysplastic syndrome, multiple myeloma, chronic lymphocytic leukaemia and haemoglobinopathies, such as sickle cell disease, and nutritional deficiencies, such as vitamin B12 and folate deficiencies [110–112]. Some medications may also decrease responsiveness to ESAs, such as proton pump inhibitors, angiotensin-converting enzyme inhibitors and/or angiotensin II receptor blockers [113, 114].

We also endorse Practice Point 3.7.6, which recommends that in patients with suspected ESA-related pure red cell aplasia (PRCA), ESA therapy should be discontinued, RBC transfusions administered as clinically indicated, and referral to a haematologist considered. Management may include immunosuppressive therapy and, after careful evaluation of risks, benefits and patient preferences, the use of a HIF-PHI for subsequent anaemia treatment.

The fact that anti-EPO antibodies can cross-react with endogenous EPO questions the additional value of HIF-PHI compared with ESA for subsequent anaemia treatment. However, some case reports showed the efficacy of HIF-PHI in this setting [115–117]. It is then possible that endogenous EPO could be less immunogenic and HIF-PHI have additional positive effects on bone marrow inflammation. Indeed, endogenous EPO is not immunogenic by itself in physiologic conditions but becomes a target of anti-EPO antibodies only after exogenous ESA administration (mainly by the subcutaneous route). Interestingly, one patient experienced PRCA remission following the shift to roxadustat in the absence of immunosuppression [117]. Also, consider kidney transplantation in suitable patients on dialysis or CKD stage 5 for the treatment of anti-EPO antibodies mediated PRCA.

## RBC TRANSFUSIONS

No major changes occurred in this section compared with the 2012 KDIGO guideline, but there is promotion of a restrictive RBC transfusion policy, especially in candidates to kidney transplantation to minimize the risk of allosensitization.

## MISSING POINTS

### Anaemia diagnosis and treatment across genders

The 2026 KDIGO guideline does not include specific recommendations regarding gender or sex differences in anaemia diagnosis, management or treatment response [1].

This represents a gap in the guideline, particularly given the emerging importance of gender medicine in general. Several aspects of anaemia in CKD are influenced by biological sex, including baseline Hb levels, iron metabolism and responsiveness to ESAs. The WHO defines anaemia using sex-specific thresholds that take into account the physiological differences between men and women [10]. Anaemia in CKD is more prevalent in females than in males [118]. In addition, females tend to have lower Hb levels than males across all stages of CKD, with the difference becoming smaller with decreasing eGFR levels [119]. Adverse health outcomes from CKD-related anaemia appear to be also more severe for females than for males [118, 120]. Clinical data show that women receiving dialysis require higher ESA doses than men to achieve similar Hb levels [121]. This observation may be due to several factors, including greater iron losses, more frequent ID and differences in ESA sensitivity. As higher doses of ESA have been linked to an increased risk of MACE in RCT, these sex-related differences could have significant clinical implications. It remains uncertain whether the same Hb targets apply to men and women during ESA/HIF-PHI therapy, considering the different Hb threshold for diagnosis. Furthermore, while large trials such as the PIVOTAL trial [5], which demonstrated the benefits of a proactive IV iron strategy, the extent to which these findings apply equally across sexes remains unexplored.

### Special considerations for pregnancy

The 2026 KDIGO guideline does not contain specific recommendations or commentary on the diagnosis and management of anaemia during pregnancy [1].

Pregnancy introduces distinct physiological changes that influence both the diagnosis and treatment of anaemia. Increased plasma volume leads to haemodilution, lowering measured Hb concentrations independent of RBC mass. At the same time, foetal and placental iron demands accelerate iron depletion, increasing the risk of absolute and functional ID. Finally, during the third trimester and puerperium, women experience a physiologically increased thrombotic risk compared with the general population—a risk that may be further amplified by ESA therapy. These changes may necessitate different diagnostic thresholds and therapeutic targets compared with the non-pregnant population. The WHO defines anaemia in pregnancy as Hb <11 g/dL at any time during pregnancy [122]. Conversely, other guidelines propose a trimester-based definition (first and third trimesters: Hb <11 g/dL, second trimester Hb <10.5 g/dL) [123, 124]. In our opinion these more precise thresholds should also be considered for pregnant women with CKD.

As indicated by obstetric guidelines [123, 124], oral iron (100–200 mg elemental iron daily) is recommended as first-line therapy for ID anaemia in pregnancy, with IV iron indicated in cases of intolerance, malabsorption or when Hb remains <10.0 g/dL after 4 weeks of oral therapy. This approach is supported by evidence suggesting that iron therapy during pregnancy—particularly IV iron administered during the second trimester—may reduce the risk of adverse outcomes, such as maternal infections, low birth weight and preterm delivery [125–127]. Of note, IV iron is contraindicated in the first trimester. No data are available on the exact definition of ID during pregnancy in CKD patients. Nevertheless, it appears to be reasonable to adopt the same criteria used for absolute ID in general for the CKD population (thus, with a higher cut-off of serum ferritin compared with the one used in the general population).

The use of ESA during pregnancy is not contraindicated; however, clinical experience is limited, and available data are insufficient to establish safety. According to regulatory labels, ESAs should be used with caution when the potential benefit to the mother outweighs the risk to the foetus. In particular, multidose vials of epoetin alfa formulations (e.g. Eprex^®^, Epogen^®^, Procrit^®^) contain benzyl alcohol, a preservative contraindicated in pregnancy and lactation due to its association with foetal and neonatal toxicity. Therefore, only preservative-free single-dose preparations should be considered in this setting.

HIF-PHIs are contraindicated in pregnancy, as they have not been studied in this population and their safety profile remains unknown.

Currently, no data are available to determine whether Hb targets should be modified during ESA therapy in pregnant women with CKD. However, given the lower diagnostic threshold for anaemia in pregnancy and the increased thrombotic risk associated with ESA therapy, aiming for lower Hb targets may be appropriate. Future research is warranted to guide evidence-based, individualized anaemia management during pregnancy in CKD [128].

### SGLT2 inhibitors, anaemia and erythrocytosis

Sodium-glucose cotransporter 2 inhibitors (SGLT2i) are indicated for the treatment of type 2 diabetes, HF and CKD [9]. Since the first clinical trials in patients with type 2 diabetes, and later in the trials in HF and CKD, these drugs produced an early and sustained increase in Hb/Hct levels [129, 130]. The rise in Hb/Hct was initially attributed to haemoconcentration from its diuretic activity and plasma volume reduction; however, later studies demonstrated early increases in EPO levels and/or reticulocyte counts—both in patients with normal and those with impaired renal function—confirming a direct stimulatory effect on erythropoiesis [129, 130].

SGLT2i also induce changes in iron biomarkers suggestive of enhanced iron metabolism. They have been shown to reduce hepcidin and ferritin levels, while some studies report increases in erythroferrone, transferrin or total iron-binding capacity (TIBC), and soluble transferrin receptor, with variable effects on serum iron concentrations and TSAT [131, 132]. All these changes are indicative of enhanced erythropoiesis and improved iron availability/utilization.

SGLT2i reduce the prevalence of anaemia and the risk of its subsequent development in patients with type 2 diabetes, with HF or with CKD [131, 132].

In patients with CKD, phase 3 trials demonstrated an average increase in Hb of approximately 0.7 g/dL and in Hct of 2.2% to 2.4% compared with placebo [133–135]. Furthermore, SGLT2i enhance the erythropoietic response to IV and oral iron in ID patients [136–138].

On the other hand, these agents may induce erythrocytosis, which may be associated with a higher thrombotic risk due to an increase in blood viscosity [139]. It is unknown whether SGLT2i may increase thrombotic risk in CKD patients with erythrocytosis, although a potential for harm cannot be entirely excluded. In the Empagliflozin Outcome Trial in Patients With Chronic HF With Preserved Ejection Fraction (EMPEROR-Preserved) trial, randomization to empagliflozin—but not placebo—was associated with a higher risk of events among patients with erythrocytosis at baseline, although the increase was not statistically significant due to the low number of events [140].

Notably, in a post-hoc pooled-analysis of the CANagliflozin Cardiovascular Assessment Study (CANVAS) program and Canagliflozin and Renal Events in Diabetes with Established Nephropathy Clinical Evaluation (CREDENCE) trials, baseline Hct levels modified the treatment effect on the primary outcome, in men but not in women [141]. Moreover, the analysis showed possible harm in men with erythrocytosis, mainly driven by an increased risk of myocardial infarction [141]. Recently, a retrospective study showed that erythrocytosis occurred in 16.9% of the patients receiving SGLT2i, with those with CKD having a reduced risk [142]. Arterial thrombosis was a rare event (0.5%, 33 patients) and was not directly attributable to SGLT2i-induced erythrocytosis [142]. Another large retrospective study of 137 552 adults with type 2 diabetes did not show any association between new-onset erythrocytosis following SGLT2i and increased risk of myocardial infarction, venous thromboembolism or stroke [143]. Similar results have been reported in patients with HFrEF [144]. The only exception comes from a retrospective cohort study using records from over 50 US healthcare organizations comparing data of patients with diabetes receiving different antidiabetic agents [145]. SGLT2i users developing erythrocytosis had increased incidence of thromboembolic events compared with those without.

Overall, the safety of SGLT2i in CKD patients with erythrocytosis deserves further studies. Associated polycythaemia vera should be ruled out in this setting since patients with this condition are at high thrombotic risk [146]. The disease should be suspected in cases of Hb >16.5 g/dL in men or >16 g/dL in women, especially if accompanied by one or more of the following suggestive features: ID, leucocytosis, thrombocytosis, pruritus or splenomegaly.

### Anaemia of CKD and anti-inflammatory drugs

In CKD, chronic low-grade inflammation plays a central role not only in anaemia and ESA hyporesponsiveness but also in the development and progression of atherosclerosis, contributing to the high burden of cardiovascular morbidity and mortality in this population [147], as well to CKD progression [147]. Anti-inflammatory therapies have been developed in CKD to target the persistent inflammatory milieu and reduced the accelerated progression of cardiovascular disease. Anti-inflammatory therapies may also reduce the prevalence of anaemia and increase Hb in anaemic CKD patients. In a post-hoc analysis of the Canakinumab Anti-inflammatory Thrombosis Outcomes Study (CANTOS), canakinumab—a monoclonal antibody targeting interleukin (IL)-1β—reduced the incidence of anaemia and increased Hb levels by 1.13 g/dL among patients with baseline anaemia, compared with placebo, in individuals with a history of myocardial infarction and elevated high-sensitivity CRP (hsCRP) [148].

Ziltivekimab, a fully human monoclonal antibody targeting IL-6, significantly reduced inflammation, ESA requirements and ESA resistance index, while increasing serum iron, transferrin and TIBC in HD patients with the TMPRSS6 rs855791 polymorphism in a phase 1–2 placebo-controlled trial [149]. Similarly, in an exploratory analysis of the Trial to Evaluate Reduction in Inflammation in Patients With Advanced Chronic Renal Disease Utilizing Antibody‑Mediated IL‑6 Inhibition (RESCUE), ziltivekimab significantly increased Hb, serum iron, TIBC and TSAT after 12 weeks in patients with CKD stages 3–5 and systemic inflammation, compared with placebo [150].

Clazakizumab, another humanized anti-IL-6 monoclonal antibody, was associated in a post-hoc analysis of the phase 2b trial (POSIBIL6ESKD) with reduced need for ESA initiation or dose escalation in dialysis patients with cardiovascular disease and/or diabetes and increased hsCRP [151]. It also increased TSAT, serum iron and TIBC, and reduced hepcidin levels [151].

These results support the role of inflammation in the anaemia of CKD and the potential use of anti-inflammatory therapies, especially among patients with ESA resistance. They are not available yet for clinical use.

## CONCLUDING REMARKS

The 2026 KDIGO Guideline for Anemia in CKD provides a substantial update from the 2012 edition, following the availability of new evidence in IV iron strategies, with greater emphasis on proactive iron repletion approaches. Following the availability of some HIF-PHI molecules for clinical use, the guidelines include a section addressing this class of anti-anaemic agents. The document reinforces that anaemia care should be individualized, patient-centred and integrated within overall CKD management.

From a European perspective, the 2026 KDIGO guideline presents certain challenges, including several unanswered questions and a lack of indication for specific patient groups. These limitations are due to the lack of strong evidence in the field in many aspects, particularly regarding long-term safety of HIF-PHIs, applicability of trial data to broader patient populations and optimal iron treatment thresholds. Safety considerations with ESAs and HIF-PHIs in certain patient subgroups highlight the need for individualized therapeutic approaches.

These guidelines represent a step forward in evidence-based nephrology practice, providing European clinicians with updated guidance for managing CKD-associated anaemia.

## Data Availability

No new data were generated or analysed in support of this research.
