# Correction to: KDIGO 2026 Clinical Practice Guideline for Anemia in Chronic Kidney Disease (CKD): a commentary from the European Renal Best Practice (ERBP)

**DOI:** 10.1093/ndt/gfag192

**Published:** 2026-09-21

This is a correction to: Lucia Del Vecchio, Aleix Cases, Michele F Eisenga, Jolanta Małyszko, Jonathan Barratt, Davide Bolignano, KDIGO 2026 Clinical Practice Guideline for Anemia in Chronic Kidney Disease (CKD): a commentary from the European Renal Best Practice (ERBP), *Nephrology Dialysis Transplantation*, Volume 41, Issue 8, August 2026, Pages 1554–1572, https://doi.org/10.1093/ndt/gfag014

In the originally published version of this manuscript, the entries in Table 2 concerning Vadadustat contained errors under the ‘FDA’ and ‘Commercial availability’ headings.

The entry in the ‘FDA’ column has been corrected to read:

‘APPROVED For DD-CKD patients only’

Instead of:

‘REJECTED Safety concerns regarding thromboembolic events; severe drug-induced liver injury reported in phase 2 trials’

In the ‘Commercial availability’ column, the section pertaining to the USA has been corrected to read:

‘USA: available for DD-CKD patients’

Instead of:

‘USA: not available’.

Additionally, under the ‘Treatment initiation and ESA maintenance therapy’ heading, text about Vadadustat approval has been corrected to read:

‘Vadadustat was initially rejected by the FDA due to safety concerns but was subsequently approved for dialysis patients in 2024. Vadadustat is also approved by the EMA for dialysis patients, and it is commercially available in several European countries’.

Instead of:

‘Vadadustat has been rejected by the FDA due to safety concerns regarding thromboembolic events and a case of severe drug-induced liver injury reported in a phase 2 trial. Conversely, it has been approved by the EMA for dialysis patients, and it is commercially available in several European countries’.

